# Peer support service activity prevalence by setting: a nine-state survey of peer workers

**DOI:** 10.3389/fpubh.2025.1533051

**Published:** 2025-03-05

**Authors:** Angela Hagaman, Hannah L. Warren, Ruth Miller, Craig Henderson

**Affiliations:** ^1^College of Public Health, East Tennessee State University, Johnson City, TN, United States; ^2^Department of Psychology and Philosophy, Sam Houston State University, Huntsville, TX, United States

**Keywords:** peer support, recovery supports, substance use disorder, opioid use disorder, drug abuse and addiction, SUD treatment, peer workforce, peer worker

## Abstract

**Introduction:**

Peer recovery support services (PRSS) are flexible, evidence-informed interventions that can be provided in a variety of settings and are delivered by credentialed people with lived-experience of mental health and substance use disorders. PRSS are a promising intervention that may increase linkage to care, treatment retention, and long-term recovery; however, there remains a sizable gap in the literature to disseminate these services to scale. Misunderstanding of the peer worker role, and a lack of consistent nomenclature to describe PRSS activities are barriers to studying PRSS effectiveness.

**Methods:**

This sequential exploratory mixed-methods study began with a qualitative and methodological review of a previous peer worker survey instrument by eight subject matter experts with lived experience of substance use disorder. The improved 38-item web-based survey was then disseminated to a non-probability sample of peer workers in nine U.S. states.

**Results:**

A total of 659 peer workers responded to the survey indicating that they perform an average of 24 different service activities most of the time (*M* = 23.6; SD = 16.7). PRSS interventions were most commonly delivered in communities and neighborhoods, client homes, and recovery community organizations. Survey participants reported spending approximately half of their time (*M* = 43.1, SD = 26.1) providing Emotional support, and less than one quarter of their time providing Affiliational (*M* = 21.3%; SD = 18.5), Informational (*M* = 18.0%; SD = 15.5), and Instrumental (*M* = 15.0%; SD = 15.3) support.

**Discussion:**

This study may be the first of its kind to explore the broad array of service activities peer workers perform in multiple settings across regional service networks, also known as recovery ecosystems. Notably, peer worker respondents selected an average of 24 activities that they perform most of the time, and Emotional support was the most commonly delivered support type. Study results provide preliminary evidence about where PRSS are performed within both macro and micro settings indicating that services are frequently delivered in non-clinical community-based settings and client homes which may confer added benefit. These results can be used to inform future studies that examine the effectiveness of PRSS across the continuum of care.

## Introduction

It is estimated that 48.5 million people in the U.S. are living with a substance use disorder (1). Overdose deaths, driven by illicit fentanyl, reached an all-time high in 2023, exceeding 112,000 in a 12-month period (2). Encouraging new estimates from 2024 suggest that overdose deaths may be on the decline and decreasing significantly in some areas of the country; and yet, overdose is still the leading cause of death among Americans between ages 18–25 years (3). Every 4 and ½ minutes a drug-related death occurs in the U.S. despite increases in federal funding to control the illicit fentanyl supply and policy-level changes that improve access to opioid overdose reversal medications (4). Moreover, certain populations, such as older Black men (5) and Native Americans (6), are now at even greater risk than ever before.

There are currently more than 17,000 drug treatment programs in the U.S.; however, limited regulations, inconsistent treatment protocols, and stigma against effective medications are leaving many vulnerable (4). Medications for opioid use disorder (MOUD) are considered the gold standard for treating opioid use disorder (OUD) and are proven to save lives and increase treatment retention, however most physicians still do not prescribe them (7). Stigma among healthcare workers remains a significant barrier to engaging persons with a substance use disorder (SUD) in treatment (8, 9), and specific strategies for linkage to care among high-risk, underrepresented populations is still widely misunderstood (10). Fewer than 1 in 5 individuals who need specialty treatment for SUD receive care (11), and many people who have an SUD do not believe they need treatment (12).

Despite the exceeding challenges in doing so, over 22 million U.S. adults have resolved a past substance use concern, and some do so without formal assistance (13, 14). Recovery from an SUD occurs through multiple pathways, and most people who engage in services do so within communities where they live and work (14, 15). Regional SUD treatment and recovery service networks, also known as “recovery ecosystems,” contain a wide variety of programs from harm reduction to clinical treatment and recovery support; however, people who may benefit most from these services may not know about them, and even if they do, the complexity of navigating the service continuum can be challenging (15, 16). In the U.S., there have historically been two well-known types of helpers supporting persons seeking recovery from SUD: the clinical addiction service provider and the mutual-aid sponsor with lived experience who has been well recognized as serving in Twelve-step recovery and other mutual-aid groups (17–19). A new helper has recently emerged following the 2007 Centers for Medicare and Medicaid Services letter to state Medicaid offices approving reimbursement for peer recovery support services (PRSS) provided by credentialed peer workers (20). Georgia was the first state to initiate reimbursement for PRSS and most states have now followed suit (21).

PRSS, as defined here, are flexible, evidence-informed interventions that can be provided in a variety of settings over varying lengths of time and are delivered by credentialed people in long-term recovery from substance use and mental health disorders (22, 23). Peer-delivered interventions are focused on supporting individuals through the recovery process and across each stage of behavioral change. Because they are available across multiple settings, they may provide distinct benefits, however, to date, methodologically rigorous research verifying the effectiveness of these interventions is limited due in part to highly variant descriptions of job titles, service activities, and settings (11, 24–27). The peer worker role remains widely misunderstood, serving as a barrier to studying its effectiveness. Recent studies have demonstrated positive effects from PRSS interventions (11, 24, 25), but the most rigorous studies are focused on opioid use disorder (OUD) exclusively and are limited to clinical treatment or hospital settings such as emergency departments, which may disproportionately exclude vulnerable populations less likely to seek medical treatment (11). It is also not well-understood how peer workers serve to improve and facilitate client navigation of services within their respective recovery ecosystems (28, 29). PRSS are intentional, person-driven approaches that center recovery goals at the individual level, and thus, may not be easily evaluated through standard clinical criteria such as abstinence, treatment adherence, or symptom reduction (30, 31).

PRSS have broad support in the U.S. and the Biden-Harris administration called for expanded access to peer support as a component of the 2022 Presidential Unity Agenda (32). The Substance Abuse and Mental Health Services Administration (SAMHSA) has released numerous resources on the implementation of PRSS including the recently released National Model Standards for Peer Support Certification (33). SAMHSA defines PRSS support across four distinct categories: Emotional (demonstrating empathy, caring, or concern to bolster self-esteem and confidence), Informational (sharing knowledge and information and/or providing life or vocational skills training), Instrumental (providing concrete assistance to help other accomplish tasks), and Affiliational (facilitating contacts with others to promote learning of social and recreational skills to acquire a sense of belonging) (34). In addition to providing individualized support to patients and clients, peer workers serve as valuable members of interdisciplinary treatment teams promoting non pejorative language among clinical staff and demonstrating what recovery can look like, thereby reducing stigma and promoting more positive attitudes toward persons with SUD (35). PRSS are implemented throughout the continuum of care and peer workers may fill distinct gaps due to behavioral health workforce shortages (23, 36).

While PRSS are a promising intervention that may increase linkage to care, motivation to change, treatment retention, and long-term recovery, there remains a sizable gap in the empirical literature to effectively disseminate PRSS to scale (11). Furthermore, as minority and marginalized populations share a disproportionate burden of SUD related consequences, PRSS delivered by peer workers at the intersection of race, ethnicity, gender, sexual orientation, gender identity, and key life experiences may confer added benefit to these populations (23). Misunderstanding of the peer role, and a lack of a consistent nomenclature to describe PRSS activities that occur across multiple service settings is a barrier to studying PRSS effectiveness. The present study engages peer workers in nine U.S. states to identify and classify common work activities and the service settings in which they are delivered, thereby pinpointing salient interventions for future study.

## Materials and methods

### Procedure

This sequential exploratory mixed-methods study began with a qualitative and methodological review of a previous peer worker survey instrument and corresponding results (27). An 8-member panel of subject matter experts assessed the quality of the existing survey instrument during a 30-day review period by providing an extensive critique on a pre-populated web-based feedback form. All experts had lived experience with SUD and included recovery scientists, peer workers, doctoral students, and expert psychometricians. Each expert provided feedback to improve the validity and reliability of the new survey instrument. Upon completion, the final quantitative survey was programmed into the Qualtrics survey platform and approved by the East Tennessee State University Internal Review Board (IRB) in February of 2023.

The final web-based peer worker survey contained 38 questions that followed an approved electronic informed consent document. Three additional prompts separate from the survey responses allowed participants to leave their email address if they were interested in a gift card lottery, participating in future research, and receiving a final copy of the study results. The web-based survey link was disseminated by email to a non-probability-based sample of peer workers by certification boards and peer associations in nine U.S. states. Participating states were selected from four US sub-regions (Northeast, Midwest, South, West) based on substance-related risk factors (treatment admissions, overdose death rates, and treatment access) and willingness to participate in survey dissemination. To be included in the study, each identified state had to have a ready champion willing to disseminate the survey email followed by two email reminders to a minimum sample of 200 certified and employed PRSS. When an identified high-risk state did not have a champion or adequate sample frame, the next “riskiest” state was then selected. Additionally, a Central Appalachian sample was utilized as the “fifth” sub-region via email addresses from the previous sample of peer workers who indicated a willingness to participate in future research (*n* = 423). The resulting final list of states in this sample included Florida, Kentucky, North Carolina, New Mexico, Pennsylvania, Tennessee, Virginia, Wisconsin, and West Virginia. Eligibility criteria required that participants be 18 years of age or older, hold a valid state, national or international peer certification, and be employed or previously employed as a peer in one of the nine states for at least 12-months. The survey link remained open for 30-days.

### Measures

As a result of the qualitative and methodological review of the original survey instrument, the list of PRSS activities from which peer workers would choose expanded from the original 14 derived from the work of Ashford et al. (15) to 62 to adequately capture the full range of services peer workers provide (see Supplementary Material A). A major factor in the expansion of the items was the large number of participants who selected “Other” in the previous version of the survey (27). Therefore, an additional 48 activities informed by the existing literature, state and national training competencies, and the open-ended responses provided by peer workers in the previous survey were included.

A similar approach was used to generate a more comprehensive list of employer types and service settings (see Supplementary Material B). The previous iteration of the peer worker survey included 15 service settings (15), and the updated instrument included both macro settings (*n* = 8)—referring to general organization and agency types—and micro settings (*n* = 28) —that is, settings in which specific activities frequently take place (e.g., syringe services programs, jails, client homes, and online or digital platforms).

### Peer certification and training variables

The survey opened with questions related to employment status, case load, and additional training or certifications (NCPRSS, NCPS, IC&RC, and CCAR). Participants were also asked about administrative and clinical supervision requirements at their current place of employment and the extent to which their state certification training prepared them for their work in the field.

### Employer and peer work activity variables

The next set of questions queried employer type from eight general/macro settings: healthcare, behavioral health/community mental health, education, not for profit/community organization, justice system, faith-based entity, government agency, and treatment clinic or agency. Additional questions collected information about a job description, and client population by age group served. Participants were also asked to estimate the average amount of time spent conducting each of the four SAMHSA-defined types of peer support: Emotional, Informational, Instrumental, and Affiliational (34). A case definition and example for each type of support was provided in the survey prompt. Another question derived from a previous study of peer workers in Michigan assessed job satisfaction (37).

Specific activities in which the peer workers were engaging was assessed by the following item: “*This next question is one of the most important in the survey. The question has 62 work activities that peer workers like yourself have asked us to include in this survey. Please read and scroll through all of the choices carefully and select ALL that apply.* After selecting all of the activities that they performed most of the time, survey skip logic provided two carryforward questions for each selection requesting that the participant select both a general/macro and then specific/micro setting in which the activity was performed. A comprehensive list of items included in this survey prompt are included in the Supplementary Material and contains 62 distinct service activities, eight general/macro settings, and more than 30 unduplicated specific/micro settings.

### Personal history and demographic variables

Survey items related to participant demographics and their personal history with recovery included type of recovery (mental health, SUD, or both), duration of recovery, criminal justice involvement, and the following demographic variables: gender identity, race, ethnicity, age, and educational level attained.

### Data analysis

Descriptive analyses were conducted on each variable using SPSS 29.0.2.0 including measures of frequency distribution, central tendency, and data set variability. To determine how many total times each specific/micro setting was endorsed, a new variable was coded for each response option (e.g., community/neighborhood) and frequencies were run on each new, re-coded variable which corresponded to each specific/micro setting. Next, data were then stratified by setting using SPSS. Using this stratification, frequency analyses were conducted for activities separately within each general/macro setting, and their subsequent specific/micro settings. The two most common general/macro settings, and three most common specific/micro settings, were examined for each of the 62 activities.

## Results

### Demographic characteristics

A total of 659 peer workers responded to the web-based survey. Seventy-six (76%) percent of respondents identified as White Non-Hispanic, 10% Black, 7% Native American/Alaskan Indian, 5% Asian, 2% Native Hawaiian or Pacific Islander, and 4% identified as another or “Other” race. Twenty-six percent (26%) also identified as Hispanic. The average age of respondents was 43 years (*M* = 43.8, SD = 13.1); 54% were female identifying, 41% were male identifying, and 4% identified as a gender other than male or female. Ninety-two percent (92%) had a high school diploma or higher level of education. Table 1 includes a comprehensive list of demographic characteristics for survey respondents and total participants by state. New Mexico (*n* = 158) had the highest number of respondents while West Virginia (*n =* 25) had the fewest.

**Table 1:** Participant demographic characteristics (*N* = 659).

Variable	*N*	Percent
State(s) peer is working in (select all that apply)		
New Mexico	158	23.90%
Wisconsin	136	20.50%
Florida	118	17.80%
Pennsylvania	97	14.70%
North Carolina	81	12.20%
Virginia	52	7.90%
Tennessee	32	4.80%
West Virginia	26	3.90%
Other State	7	1.10%
Race and Ethnicity (select all that apply)		
White/Caucasian	432	75.90%
Black or African American	57	10.00%
Native American, Alaskan, or American Indian	40	7.00%
Asian or Asian American	28	4.90%
Another race	20	3.50%
Native Hawaiian or Pacific Islander	9	1.60%
Non-Hispanic	403	71.70%
Hispanic	146	26.00%
Prefer not to answer	13	2.30%
Age		
26–44	249	50.80%
45–64	178	36.30%
18–25	35	7.10%
65 or older	28	5.7
Present gender identity		
Female	310	54.40%
Male	234	41.40%
Other gender identity	17	3%
Transgender	6	1.10%
Prefer not to answer	3	0.50%
Education level		
BA or BS degree	144	25.30%
Associate degree	136	23.90%
High-school diploma	134	23.50%
Graduate degree	108	18.90%
GED	39	6.80%
None	5	0.90%
Prefer not to answer	4	0.70%
Recovery type		
Both SUD and a mental health disorder	271	47.50%
Mental health disorder	155	27.20%
Substance use disorder (SUD)	96	16.80%
Prefer not to answer	48	8.40%
Years in recovery		
0–2	168	29.20%
10 or more	156	27.10%
03-May	126	21.9.%
06-Oct	126	21.90%

### Training, employment, and peer certification

Sixty-seven percent (67%, *n* = 439) of survey respondents had a state level certification. Additionally, 216 had the NAADAC Certified Peer Recovery Support Specialist credential (NCPRSS), 197 had the IC&RC Peer Recovery credential, 196 had the Mental Health America National Certified Peer Specialist credential (NCPS), and 173 had the Connecticut Community for Addiction Recovery (CCAR) Recovery Coach credential. Ninety-four percent (94%) of respondents were employed at the time of survey dissemination, with an average of 6 years working as a professional in the addictions field (*M* = 6.2, SD = 7.3). Most of the peer workers in the sample (87.0%) had fewer than 50 clients on their active caseload and reported working an average of 35 h per week (*M* = 34.8, SD = 14.0). Seventy-seven percent had an established job description, and 76% were required to receive clinical or administrative supervision as a component of their job or peer certification requirements. However, only 20% received this supervision from another credentialed peer. Peer worker respondents reported that they were satisfied or very satisfied with most features of their job including physical safety (89%), job security (83%), and other peer staff supportiveness (78%); however, fewer were satisfied with supervisor supportiveness (70%), work-related stress (69%), and promotion opportunities (65%). Regarding hourly wages, 39% reported an hourly wage of $16–$20 dollars, 25% made more than $20 per hour, and 22% made $10–$15 per hour. Fewer than 3% reported making less than $10 per hour. Table 2 includes additional detail on these results.

**Table 2:** Peer training and certification (*N* = 659).

Variable	*N*	Percent
Certification(s) held (select all that apply)		
State-level PRSS certification(s)	439	67.10%
NAADAC National Certified Peer Recovery Support Specialist (NCPRSS)	216	39.70%
Peer Recovery Credential with the International Certification and Reciprocity Consortium (IC&RC)	197	36.80%
Mental Health America’s National Certified Peer Specialist (NCPS)	196	36.40%
Connecticut Community for Addiction Recovery (CCAR) Recovery Coach	173	32.30%
Other national or international certification	111	31.8
Length of time peers have worked in their current job		
1 year or less	155	28.40%
2–5 years	292	53.60%
6–10 years	71	13.00%
11–15 years	14	2.60%
16–19 years	6	1.10%
More than 20 years	7	1.30%
Peer supervision requirement and supervisor type		
Supervision required (Yes)	503	77.10%
Supervision not required (No)	149	22.90%
Behavioral health or clinical staff provides supervision	247	50.50%
Non-clinical staff or supervisor provides supervision	113	23.10%
Certified peer provides supervision	100	20.40%
Someone else provides supervision (Other)	29	5.90%
Primary age group peers are working with		
Adults (27–64)	478	73.80%
Young Adults	107	16.50%
Other age group	43	6.60%
School aged children (k-12 or equivalent age)	10	1.50%
Seniors (65+)	10	1.50%
Typical number of clients/patients peers work with		
0–25	284	49.30%
26–50	217	37.70%
51–75	45	7.80%
76–100	15	2.60%
More than 100	15	2.60%
Extent to which peers feel their certification(s) and training(s) prepared them for their current job		
A great deal	337	51.50%
Some	255	39.00%
Very little	60	9.20%
Not at all	2	0.30%

### Work activities and settings

Most of the peer worker respondents were employed by a behavioral health/community mental health agency (40%) or a not-for-profit community organization (27%) followed by treatment clinics (9%) and healthcare organizations (9%). Fewer than 20% were working in the justice system, education, faith-based entities, or government agencies combined. When asked what percent of their work time was spent in each of the four types of peer support as defined by SAMHSA, respondents reported spending nearly half of their time providing Emotional support (*M* = 43.1%; SD = 26.1), followed by Affiliational (*M* = 21.3%; SD = 18.5), Informational (*M* = 18.0%; SD = 15.5), and Instrumental support (*M* = 15.0%; SD = 15.3).

**Figure 1:**
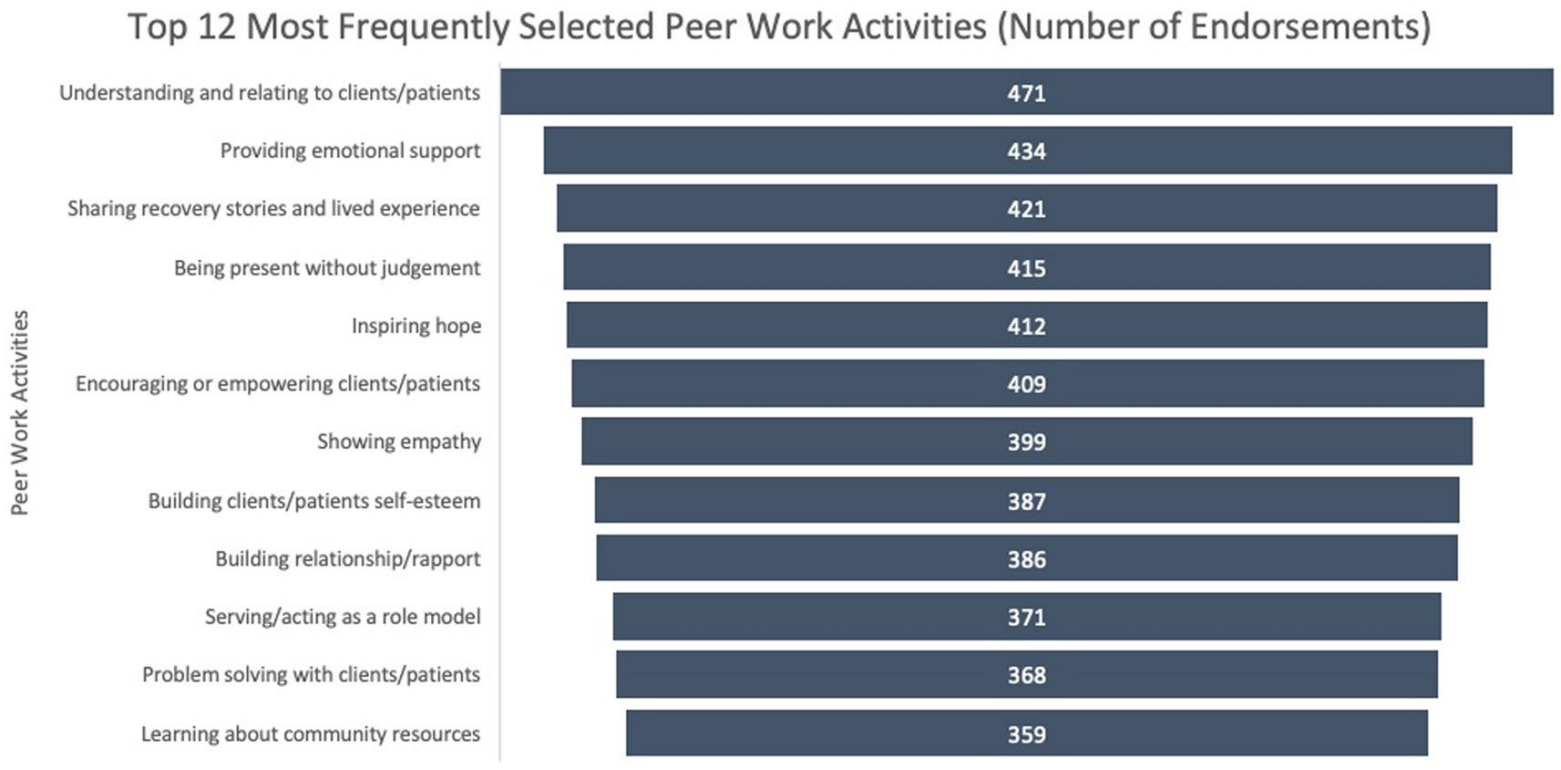
Number of participants who endorsed each of the top 12 most frequently selected activities from the total list of 62 discrete work activities.

When asked to select activities that they performed most frequently, respondents selected 24 activity choices on average (*M* = 23.6, SD = 16.7) from the list of 62 discrete activities. As illustrated in Figure 1, peer workers in this sample most frequently endorsed activities that could be categorized as Emotional support (e.g., understanding and relating to clients, providing emotional support, sharing recovery stories, being present without judgment, inspiring hope, encouraging or empowering, showing empathy, etc.).

**Figure 2:**
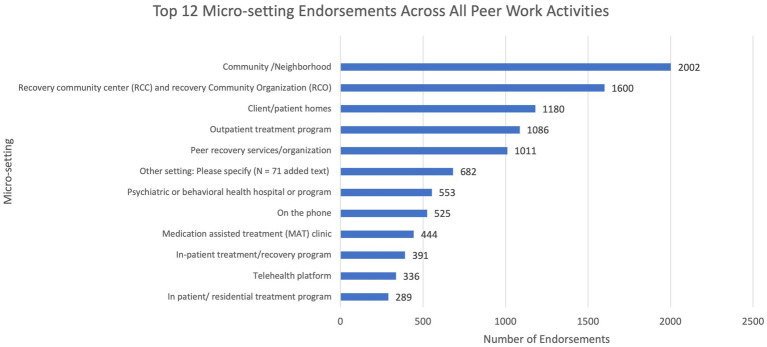
Most common micro-setting endorsements (the actual location where the work activity is performed) for all 62 discrete work activity selections.

The nested general/macro and specific/micro setting prompts that followed each activity endorsement indicate that the general/macro settings associated with each activity closely follow the employer by type results: the majority of endorsed activities occur in either not-for-profit/community organization (32%) or behavioral health/community mental health (31%) general/macro settings. However, the specific/micro settings where activities were performed had greater variance. Figure 2 illustrates this variability, with most activity endorsements occurring in communities and neighborhoods where patients/clients live and not in clinical or agency settings.

Table 3 provides further detail outlining the nested nature of survey responses and corresponding results. When PRSS respondents selected an activity, they commonly reported performing that activity in either a behavioral health/community mental health agency or a not-for-profit organization macro/general setting. Yet, a wider variety of specific/micro settings within these two general/macro categories were selected. For instance, respondents most commonly endorsed performing the “understanding and relating to clients” activity in a peer recovery services organization, yet they most commonly performed the “building self-esteem” activity in a community/neighborhood setting. Finally, community/neighborhood settings account for the greatest number of specific/micro setting endorsements (*n =* 2002, 15.2%), followed by recovery community centers/recovery community organizations (*n =* 1,600, 7.1%). Table 4 illustrates frequency and percent for all specific/micro setting endorsements.

**Table 3:** Top four most frequently selected activities by macro and micro settings.

Activity	Settings where activities are most frequently performed	
	General/Macro setting	Specific/Micro setting
1. Understanding or relating to clients or patients (*N* = 471)	Behavioral health/community mental health (*N* = 156)	Outpatient treatment program; community/neighborhood (*N* = 26)
		Psychiatric or behavioral health hospital or program (*N* = 25)
		Client homes (*N* = 20)
	Not for profit/community organization (*N* = 134)	Peer recovery services/organization (*N* = 28)
		Community/neighborhood (*N* = 15)
		Recovery community organization (RCO; *N* = 14)
2. Providing emotional support (*N* = 434)	Behavioral health/community mental health (*N* = 139)	Community/neighborhood (*N* = 23)
		Outpatient treatment program (*N* = 22)
		Psychiatric or behavioral health hospital or program (*N* = 21)
	Not for profit/community organization (*N* = 129)	Peer recovery services/organization (*N* = 28)
		Recovery community organization (RCO; *N* = 19)
		Community/neighborhood (*N* = 14)
3. Sharing recovery stories and lived experience (*N* = 421)	Behavioral health/community mental health (*N* = 128)	Outpatient treatment program (*N* = 23)
		Psychiatric or behavioral health hospital or program; Community/neighborhood (*N* = 22)
		Client homes (*N* = 13)
	Not for profit/community organization (*N* = 119)	Peer recovery services/organization (*N* = 30)
		Community/neighborhood; Recovery community organization (RCO); (*N* = 15)
		Recovery Community Center (RCC; *N* = 8)
4. Being present without judgment (*N* = 415)	Not for profit/community organization (*N* = 137)	Peer recovery services/organization (*N* = 26)
		Community/neighborhood; Recovery community organization (RCO; *N* = 16)
		Client/patient homes (*N* = 12)
	Behavioral health/community mental health (*N* = 130)	Community/neighborhood (*N* = 23)
		Outpatient treatment program (*N* = 21)
		Client homes (*N* = 20)

**Table 4:** Micro-setting endorsements across all peer work activities (*N* = 659).

Variable	*N*	Percent
Micro-setting selection (select all that apply)		
Community /neighborhood	2002	15.20%
Recovery community center (RCC) or organization (RCO)	1,600	7.10%
Client/patient homes	1,180	8.90%
Outpatient treatment program	1,086	8.20%
Peer recovery services/organization	1,011	7.60%
Other setting: Please specify (N = 71 added text)	682	5.10%
Psychiatric or behavioral health hospital or program	553	4.20%
On the phone	525	4.00%
Medication assisted treatment (MAT) clinic	444	3.40%
In-patient treatment/recovery program	391	3.00%
Telehealth platform	336	2.50%
In patient/ residential treatment program	289	2.20%
Reentry service	283	2.20%
Homeless shelter or service organization	246	1.90%
Housing or recovery residence	222	1.70%
County government	221	1.70%
Crisis intervention/response center	206	1.60%
Veterans Administration (VA) Hospital/Clinic	182	1.40%
Recovery residence/transitional housing	166	1.30%
Overdose response services	163	1.20%
Advocacy organization	137	1.00%
Peer respite service	131	1.00%
Online messaging or application	104	0.80%
Anti-drug coalition	100	0.80%
County jail	92	0.70%
Prevention program	88	0.70%
State government	82	0.60%
Managed care organization (MCO)	79	0.60%
Post-incarceration re-entry program	70	0.50%
Hospital emergency department (ED)	69	0.50%
Collegiate recovery program	68	0.50%
Faith-based treatment program	65	0.50%
Prison	59	0.40%
Harm reduction/syringe service program	58	0.40%
Community corrections	54	0.40%
Recovery/drug court	53	0.40%
Specialty care office	51	0.40%
Federally qualified health center (FQHC)	50	0.40%
Church or congregation	48	0.40%
In-patient hospital-based detox or treatment program	47	0.40%
Forensic unit	42	0.30%
Mutual aid organization (AA,NA,etc.)	42	0.30%
Primary care office	41	0.30%
Health department	40	0.30%
Detoxification program	39	0.30%
Recovery high school	38	0.30%
K-12 School system	35	0.30%
Hotline or crisis call center	34	0.30%
College or university	32	0.20%
Acute care general hospital	31	0.20%
Recovery residence/dorm	29	0.20%
Sobering center	27	0.20%
Law enforcement agency	25	0.20%
Parole/probation program	25	0.20%
Child and family services	21	0.20%
Domestic violence organization	21	0.20%
Student homes	21	0.20%
Faith-based housing program	20	0.20%
Social services	14	0.10%
Federal government	9	0.10%
Health insurance agency	9	0.10%
HIV/AIDS health center	5	0.00%
Pharmacy	5	0.00%
Dental clinic or program	3	0.00%
Emergency medical services (EMS)	3	0.00%
Faith-based transportation program	2	0.00%
Total	13,248	100.00%

### Personal history

Peer worker respondents (*N* = 659) had an average of 9 years in recovery (*M* = 9.3, SD = 8.5). Forty-four (44%) percent had previous criminal legal system involvement, and 41% reported being in recovery from both an SUD and a mental health condition. Only 15% were in recovery from SUD alone, while 23% were in recovery from a mental health condition alone.

## Discussion

This study addresses a universal call in the literature for clarity in describing PRSS interventions, the peer worker role, and a uniform classification of service activities that can serve as a foundation for effectiveness research. Cited barriers to conducting effectiveness studies include highly variant titles for peer workers, a broad range of interventions conducted in numerous settings, outcome measures across all types of SUD, and disaggregation of the effects of PRSS from other types of treatment and recovery services (11, 22, 26). It is hypothesized that PRSS may lead to a broad array of proximal and distal outcomes that may extend beyond SUD treatment adherence, reduction in substance use, and long-term recovery (29). A comprehensive classification of PRSS activities and settings in which they are performed does not yet exist for this relatively new workforce.

The present study examined survey results from 659 peer workers in nine U.S. states and may be the first of its kind to explore the broad array of service activities peer workers perform in multiple settings across regional recovery ecosystems. Notably, peer worker respondents selected an average of 24 discrete activities that they perform most of the time. These results also provide preliminary evidence about where service activities are performed within both general/macro and specific/micro settings.

Peer workers reported that they spend about half of their time providing Emotional support and less than ¼ of their time providing each of the other types of SAMHSA-defined peer support (Informational, Instructional, and Affiliational). These results support findings from a previous study in which Central Appalachian peer workers (*N* = 565) reported spending 52% of their time providing Emotional support (27). Recommendations for future research would include an exploration of the manner in which these Emotional support activities are delivered and the underlying mechanisms of action that may lead to positive outcomes.

These study results also indicate that PRSS interventions are delivered in neighborhoods, client homes, and recovery community organizations more frequently than in clinical treatment settings. It is hypothesized that engagement at the community level may confer specific advantages due to cited barriers to clinical treatment engagement and retention (8, 16, 38). PRSS provided in community settings may also lead to increased engagement among vulnerable communities as peer workers may be uniquely poised to engage in a culturally competent manner using their shared lived experience (29, 35). Peer workers in this sample also frequently provide services in peer-led organizations such as recovery community centers (RCCs) and recovery community organizations (RCOs) that offer free, low-threshold points of engagement that may be particularly appealing to persons who may be ambivalent about clinical treatment, have experienced stigma in healthcare settings (8), or who are concerned that they cannot pay for specialty treatment (16).

The inclusion of PRSS in the cascade of SUD care has gained enormous momentum in the U.S. (29), and Medicaid and third-party reimbursement for peer support has led to integration of PRSS in medical settings such as emergency departments, inpatient treatment centers, and primary care. Peer workers’ lived experience may make them more relatable, and perhaps more equipped to establish and maintain rapport, than clinical staff, which may lead to greater treatment engagement and sustainment of long-term recovery (19, 29). However, notably, peer workers are commonly paid less than other behavioral health staff, and while they are generally satisfied with their work, work related stress and career advancement are areas of concern (27). Peer workers also report feeling stigmatized by non-peer colleagues who also frequently misunderstand their role (39), and they are often asked to perform tasks that do not align with the intent of the peer model, such as providing transportation, documentation, and case management (27). Peer workers frequently seek and attain additional training and national and international certifications, but there are relatively few options for increased pay and career advancement for this workforce (27). To address the challenges faced by this emergent workforce, future studies should include peer workers in all aspects of study design and include an explicit focus on how to support and sustain this vital workforce.

### Strengths and limitations

The present study represents one of the first comprehensive accounts of the activities in which peer workers engage with respondents representing a national sample, and as such, the findings hold promise for future studies focused on the impact these activities have on substance use outcomes. Although the sample was national in scope, it was not obtained randomly, nor based on probability sampling; therefore, the generalizability may be limited to the present respondents. Future work should replicate the methods and measures but use sampling frames that could involve random selection and stratification on key variables. A second limitation was with the survey instrument itself. Although the rates of survey completion were high for research of this kind (85% of respondents completed the instrument), approximately 15% of participants exited the survey during the section on PRSS activities. Whereas we believe the comprehensive account of activities, along with general/macro and specific/micro settings, is a strength of the present study, considerations could be made for the optimal placement of these questions in the overall instrument. Finally, the results are impacted by self-report bias; however, we assume that as the questions focus on daily routines, the impact may be minimal.

## Conclusion

It is currently estimated that fewer than 1 in 5 individuals in the U.S. receive specialty treatment for their OUD or SUD (11), and many people who have an SUD do not believe they need treatment (1). PRSS activities, specifically those that offer Emotional support, may provide unique benefit when delivered by persons with shared lived experience across the continuum of care. However, not knowing specifically what peers are doing across multiple settings, has limited effectiveness research and outcome measurement. Furthermore, it is not well understood how regional recovery service settings and SUD recovery and treatment providers are supporting and sustaining this valuable workforce. This study seeks to advance the science of PRSS through the categorization of common service activities and settings among a large sample of peer workers (*N =* 659) in nine U.S. states. A future direction for this line of research will include a random sample of peer workers across the U.S. to inform the development of a formal nomenclature for PRSS activities and service settings.

## Data Availability

The raw data supporting the conclusions of this article will be made available by the authors, without undue reservation.
