# Supplementary material for: Peer support service activity prevalence by setting: a nine-state survey of peer workers
Source: Front Public Health. 2025 Mar 5;13:1533051. doi: 10.3389/fpubh.2025.1533051 (PMC11922078; doi:10.3389/fpubh.2025.1533051)
Supplement: Supplementary file 1 [file Data_Sheet_1.docx]

**Appendix A. PRSS Work Activities**

| 1. Sharing recovery stories and lived experience 2. Serving/acting as a role model 3. Understanding and relating to clients/patients 4. Providing emotional support 5. Showing empathy 6. Inspiring hope 7. Learning about community resources/maintaining a resource list 8. Providing assertive community outreach to high-risk client/patients/communities 9. Helping clients/patients make social connections and friends 10. Being present and listening to clients/patients without judgement 11. Building clients/patients self-esteem 12. Encouraging or empowering clients/patients 13. Increasing motivation for change 14. Using motivational interviewing (MI) techniques 15. Offering or providing legal advocacy 16. Providing education / building skills 17. Facilitating trainings 18. Teaching effective coping strategies 19. Conducting mental health or substance abuse screenings 20. Using contingency management techniques 21. Advocating or serving as a voice for clients/patients 22. Providing forensic or court support 23. Collecting or analyzing data 24. Reducing clients/patient’s self-stigma and shame 25. Reducing stigma among co-workers or community partners 26. Building relationship/rapport 27. Linkage facilitation/linking clients/patients to services 28. Providing trauma informed care 29. Helping clients/patients navigate systems or services 30. Identifying symptoms of physical or sexual abuse 31. Problem solving with clients/patients 32. Working with other family members 33. Helping clients/patients meet basic needs 34. Developing, leading/facilitating groups 35. Providing health and wellness support 36. Integrating physical and mental health care 37. Developing person-centered treatment plans 38. Establishing recovery goals 39. Supporting compliance with recovery court or other court mandates 40. Providing employment assistance 41. Providing post-treatment monitoring 42. Encouraging/monitoring the appropriate use of medication 43. Teaching effective communication skills 44. Teaching/monitoring relapse prevention 45. Assisting with spiritual / religious engagement 46. Providing support with co-existing behavioral health conditions (depression, anxiety) 47. Helping with housing assistance 48. Teaching life skills or daily tasks 49. Providing crisis support 50. Working with persons who have been trafficked 51. Providing harm reduction services 52. Overdose response 53. Providing case management 54. Providing benefits assistance (health insurance, disability or social security benefits, childcare assistance benefit) 55. Teaching financial literacy or financial planning 56. Participating in community visits with clients 57. Completing intake, documentation, billing, or other paperwork 58. Supervising/ training other peer workers 59. Teaching/training non-peer staff and others about recovery processes and programs 60. Collaborating with community partners 61. Building community 62. Providing transportation |
| --- |
