# Supplementary material for: Peer support service activity prevalence by setting: a nine-state survey of peer workers
Source: Front Public Health. 2025 Mar 5;13:1533051. doi: 10.3389/fpubh.2025.1533051 (PMC11922078; doi:10.3389/fpubh.2025.1533051)
Supplement: Supplementary file 2 [file Data_Sheet_2.docx]

**Appendix B.**

**PRSS General/Macro and Specific/Micro Settings**

| Please select the type of agency where you do this activity. | Please select a specific location where you conduct this activity most of the time. |
| --- | --- |
| General (Macro) | **Specific (Micro)** |
| Healthcare | Acute care general hospital  Primary care office  Specialty care office  Hospital Emergency Department (ED)  Sobering Center  In-patient Hospital-based Detox or Treatment Program  Out-patient treatment program  Psychiatric or behavioral health hospital or program  Federally Qualified Health Center (FQHC)  Pharmacy  Veteran’s Administration (VA) Hospital/Clinic  HIV/AIDS health center  Dental clinic or program  Harm reduction/Syringe Service Program  Community / Neighborhood  Patient homes  Emergency Medical Services (EMS)  Health insurance agency  Managed care organization (MCO)  Medication assisted treatment (MAT) clinic  On the phone  Telehealth platform  Online messaging or application  Other setting: Please describe _________ |
| Behavioral Health/Community Mental Health | Outpatient treatment program  Psychiatric or behavioral health hospital or program  In-patient treatment/recovery program  Recovery residence/transitional housing  Detoxification program  Crisis intervention/response center  Overdose response services  Harm reduction/Syringe Service Program  Prevention Program  Community / Neighborhood  Client homes  Reentry service  Medication assisted treatment (MAT) clinic  Hotline or Crisis Call Center  On the phone  Telehealth platform  Online messaging or application  Other setting: Please describe _________ |
| Education | K-12 School system  College or University  Collegiate recovery program  Recovery residence/dorm/transitional housing  Recovery High School  Student homes  Community /Neighborhood  On the phone  Telehealth platform  Online messaging or application  Other setting: Please describe _________ |
| Not for Profit/  Community Organization | Recovery community center (RCC)  Recovery community organization (RCO)  Peer recovery services/organization  Overdose response team  Anti-drug coalition  Peer respite service  Domestic violence organization  Homeless shelter or service organization  Harm reduction/Syringe Service Program  Prevention Program  Reentry Services  Mutual Aid Organization (AA,NA,etc.)  Advocacy Organization  Housing or recovery residence  Medication assisted treatment (MAT) clinic  Community /Neighborhood  Client/patient homes  On the phone  Telehealth platform  Online messaging or application  Other setting: Please describe _________ |
| Justice System | County Jail  Prison  Recovery/Drug Court  Community corrections  Post-incarceration re-entry program  Parole/probation program  Law enforcement agency  Forensic unit  Community /Neighborhood  Client/patient homes  On the phone  Telehealth platform  Online messaging or application  Other setting: Please describe _________ |
| Faith-Based Entity | Faith-based housing program  Faith-based transportation program  Faith-based treatment program  Church or Congregation  Community /Neighborhood  Client/patient homes  On the phone  Telehealth platform  Online messaging or application  Other setting: Please describe _________ |
| Government Agency | Social services  Child and Family Services  Child Welfare Services  Federal Government  Veterans Administration  Health Department  County Government  State Government  Community /Neighborhood  Client/patient homes  On the phone  Telehealth platform  Online messaging or application  Other setting: Please describe _________ |
| Treatment/Clinic or Agency | In patient/ residential treatment program  Out-patient treatment program  Medication assisted treatment program (buprenorphine)  Medication assisted treatment program (methadone)  Community /Neighborhood  Client/patient homes  On the phone  Telehealth platform  Online messaging or application  Other setting: Please describe _________ |
